# Prior epigenetic priming of cytokine genes in naive T cells is required for their subsequent activation by inducible enhancers

**DOI:** 10.1186/1756-8935-6-S1-O38

**Published:** 2013-03-18

**Authors:** Sally R James, Sarah L Bevington, Fabio Mirabella, Marjorie Boissinot, Euan W Baxter, Sarion R Bowers, Peter N Cockerill

**Affiliations:** 1 Leeds Institute of Molecular Medicine, University of Leeds, Leeds LS9 7TF, UK; 2 College of Medicine and Dentistry, Institute for Biomedical Research, University of Birmingham, Birmingham B152TT, UK

## 

The inducible lL-3 and GM-CSF genes can only be efficiently expressed in T cells once they have been through a previous cycle of activation and undergone a process termed blast cell transformation [1]. An initial stimulus is required to bring naive T cells out of the resting state and into the cell cycle. This process is triggered by T cell receptor (TCR) stimulation, takes about 24 hours, and is associated with extensive nuclear remodeling. Once primed by a cycle of activation, T blast cells can maintain their ability to express lL-3 and GM-CSF for many cell divisions without the continual need for additional stimuli. In contrast, the lL-3 and GM-CSF genes cannot be induced in naive T cells that have never received a TCR stimulus. We show that this pattern of regulation of the IL-3/GM-CSF locus is controlled at two distinct levels:

(**1**) During blast cell transformation, the IL-3/GM-CSF locus acquires an extensive array of DNase I Hypersensitive Sites (DHSs) which are then maintained indefinitely for many cell cycles [1,2]. These primed DHSs are marked by me2K4 histone H3, and they also persist in non-dividing memory T cells in the peripheral blood [1]. These DHSs are absent in the thymus, spleen T cells, and naive T cells in the blood. These DHSs do not function as classical enhancers, and we propose that they serve to maintain an active chromatin structure in previously activated T cells.

(**2**) The expression of the lL-3 and GM-CSF genes is in each case dependent on the activation of inducible upstream enhancers. The lL-3 and GM-CSF enhancers appear as inducible DHSs T blast cells within 20 min of stimulation, and only acquire the me2K4H3 modification after stimulation. These enhancers are dependant on the TCR inducible factors NFAT and AP-1. However, although AP-1 and NFAT family mRNAs are efficiently expressed in both naive T cells and in T blast cells, the enhancers only respond to induction of these factors in T blast cells. The inducible DHSs remain completely undetectable in naive T cells even after 4 hours of stimulation with direct activators of TCR signaling pathways (PMA and Calcium ionophore).

We propose that T blast cells and memory T cells have developed a strategy of maintaining a discrete class of DHS as epigenetic memory modules that support an accessible chromatin environment and thereby prime inducible genes for efficient re-activation when memory cells re-encounter Ag stimuli. In the absence of the priming elements, the lL-3/GM-CSF locus appears to remain inaccessible to transcription factors that are otherwise very efficient at recruiting the chromatin remodelers that create DHSs within the inducible enhancers.
